# Photoelastic Analysis of Fixed Partial Prosthesis Crown Height and Implant Length on Distribution of Stress in Two Dental Implant Systems

**DOI:** 10.1155/2014/206723

**Published:** 2014-10-08

**Authors:** Evandro Portela Figueirêdo, Eder Alberto Sigua-Rodriguez, Marcele Jardim Pimentel, Ana Regina Oliveira Moreira, Mauro Antônio de Arruda Nóbilo, José Ricardo de Albergaria-Barbosa

**Affiliations:** ^1^Department of Odontology, Unified University Center of Maranhão (UNICEUMA), 65.075-120 São Luís, MA, Brazil; ^2^Department of Oral and Maxillofacial Surgery, Piracicaba Dental School, P.O. Box 52, University of Campinas (UNICAMP), 13414-903 Piracicaba, SP, Brazil; ^3^Department of Prosthesis and Periodontology, Piracicaba Dental School, P.O. Box 52, University of Campinas (UNICAMP), 13414-903 Piracicaba, SP, Brazil

## Abstract

The aim of this study was to evaluate by photoelastic analysis stress distribution on short and long implants of two dental implant systems with 2-unit implant-supported fixed partial prostheses of 8 mm and 13 mm heights. Sixteen photoelastic models were divided into 4 groups: I: long implant (5 × 11 mm) (Neodent), II: long implant (5 × 11 mm) (Bicon), III: short implant (5 × 6 mm) (Neodent), and IV: short implants (5 × 6 mm) (Bicon). The models were positioned in a circular polariscope associated with a cell load and static axial (0.5 Kgf) and nonaxial load (15°, 0.5 Kgf) were applied to each group for both prosthetic crown heights. Three-way ANOVA was used to compare the factors implant length, crown height, and implant system (*α* = 0.05). The results showed that implant length was a statistically significant factor for both axial and nonaxial loading. The 13 mm prosthetic crown did not result in statistically significant differences in stress distribution between the implant systems and implant lengths studied, regardless of load type (*P* > 0.05). It can be concluded that short implants showed higher stress levels than long implants. Implant system and length was not relevant factors when prosthetic crown height were increased.

## 1. Introduction

Limitations regarding the volume and geometry of the alveolar bone are common in the posterior maxilla and mandible at the time of rehabilitation with dental implants. Thus, the use of short implants has been considered a therapeutic alternative in cases of unavailable bone height, since short implants adapt to the rehabilitated site anatomy and exclude the need for reconstructive surgical procedures. This approach also reduces the occurrence of surgical complications, morbidity, costs of the treatment, and treatment time [1].

The rehabilitation of severely resorbed alveolar ridges without bone reconstruction procedures requires prostheses with increased crown height so that patients' occlusion can be reestablished [2]. This is an important factor in the rehabilitation of partially edentulous patients because the greater the distance between the occlusal contact and the crestal bone around the implant, the greater the overload experienced by the implant [3, 4]; this is especially true when the implant has reduced length and is subjected to more biomechanical complications related to occlusal loads transfer due to the smaller area of bone-implant contact [5]. Because the stress depends on the intensity of the force and the area where the force is applied, it is necessary to reduce the force or increase the surface area to avoid stress-related complications [6]. Thus, efforts are focused on the factors that can decrease load transfer along bone-implant interface, such as the type of loading, implant material properties, prosthesis material properties, macrostructure of the prosthesis, geometry, and surface structure of the implant [7, 8].

Conversely, Blanes [9], in a systematic review, evaluated the influence of the crown-implant ratio in the survival rate of implant-supported reconstructions. Although the authors have observed heterogeneity among study designs and the applied methodology for data collection in the clinical trials, the results showed that the crown-implant ratio did not influence peri-implant bone loss or implant prosthetic complication rates. However, other factors may influence load transfer to bone-implant interface and therefore the crown-implant ratio should not be the only parameter evaluated to determine the impact on bone resorption, implant success rates, complication rates, and implant prosthetic [10].

Better results for rehabilitation with dental implants are achieved by increasing the number and diameter of the implants when implant-supported fixed partial prostheses are used. These conditions increase bone-implant contact area and reduce excessive occlusal forces [4, 11]. In a similar way, tapping implants are also used to increase the surface area of the implant. Thus, variations in geometry of implant threads—such as thread pitch shape, design, and height—may play an important role in the type of forces transmitted to the area surrounding the implant [4, 12].

Given the absence of studies assessing the relationship between prosthetic factors (such as prosthesis height) and implant factors (such as length and macrostructure), the present study aimed to compare the stress distribution in short and long implants of two different dental implant systems with fixed prostheses of 8 and 13 mm crown height under axial and nonaxial loading in photoelastic models. The hypothesis is that short implants present higher stress concentration, regardless of implant macrostructure and loading, especially when associated with prosthetic crown of increased height.

## 2. Material and Methods

### 2.1. Study Design

**Figure 1:**
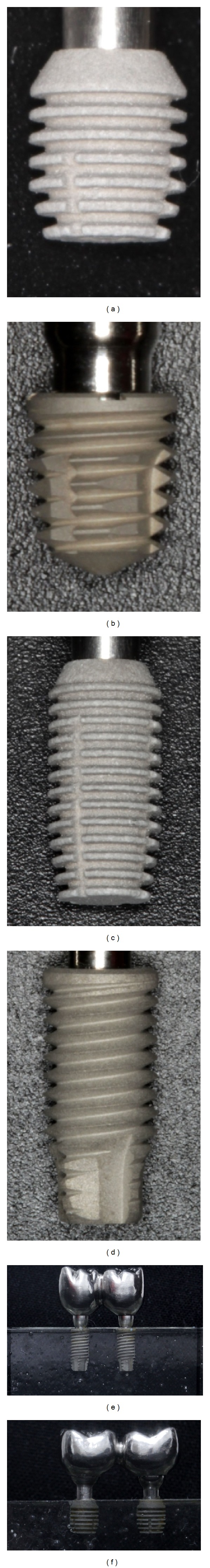
Implants and prosthetic crowns used in the study: (a) short implant (Bicon), (b) short implant (Neodent), (c) long implant (Bicon), (d) long implant (Neodent), (e) 2-unit implant-supported fixed partial prostheses of 8 mm prosthetic crowns, and (f) 2-unit implant-supported fixed partial prostheses of 13 mm prosthetic crowns.

To conduct the present study, implant length (short and long), height of the prosthesis (8 or 13 mm), and implant system (Neodent and Bicon) were considered as study factors (Figure 1). For an 80% power to detect differences among factors, it required a sample of four models for each group (SAS v. 9.2; SAS Institute, Inc., Cary, NC). Sixteen photoelastic resin models with installed implants and abutments were obtained and divided into four groups according to the implant system and implant length, with four replicates for each group (Table 1). Each replica was subjected to axial and nonaxial (15°) loading, with both 8 mm and 13 mm prosthetic crown heights. Cylindrical implants with Morse taper connection from different manufactures were tested (Neodent System and Bicon System).

### 2.2. Photoelastic Resin Models

**Figure 2:**
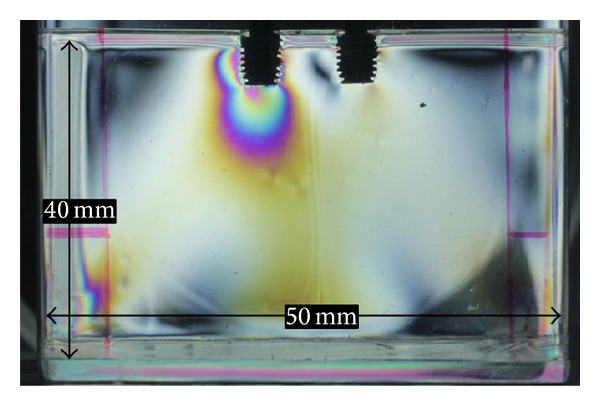
Acrylic matrix with implant.

One rectangular-shaped polished acrylic matrix was made for each group. The matrices with dimensions of height, length, and thickness were 40 × 50 × 10 mm for obtaining the resin models in the same dimensions (Figure 2).

Two perforations were performed on the upper surface of each acrylic matrix with a distance of 12 mm from center to center of each analog. The transferees were adapted to their respective implant analogs and inserted perpendicularly with the help of a parallelometer (BioArt, São Carlos, SP, Brazil) in the perforations. Two similar analog implants were installed in each perforation and retained with adhesive glue (Loctite, Itapevi, SP, Brazil). The position of the analogs was determined to simulate the clinical situation of two-element fixed prostheses.

The set acrylic matrix and transferee were placed in a plastic container and completely covered by blue silicone ASB-10 (Polipox Industry and Commerce Ltda, São Paulo, SP, Brazil) to obtain the silicon mold. The set was removed after curing time of 24 hours, according to the manufacturer's recommendations.

The transferees were adapted to their implants, and flexible epoxy resin photoelastic III (Polipox Industry and Commerce Ltd., São Paulo, SP, Brazil) was poured into the set. A period of 24 hours was needed to reach the final material and the photoelastic model was removed from the impression. Thus, a translucent, stress-free model, appropriate for the photoelastic analyses, was obtained.

Four two-element fixed prostheses were manufactured with chrome-cobalt alloy (StarLoyC Degudent, Dentsply, Petropolis, RJ, Brazil) by conventional technique. Prosthetic crowns were made over the abutments positioned in the acrylic matrices. Two prostheses had 13 mm height, 10 mm mesiodistal length, and 8 mm buccolingual width. The other two prostheses had 8 mm height, 10 mm mesiodistal length, and 8 mm buccolingual width (Figures 1(e) and 1(f)).

To perform the analysis of the stress induction, the prostheses were cemented in the photoelastic model. After that, models were observed in the polariscope to verify the absence of residual stresses, which might interfere with fringe analysis.

### 2.3. Loading

To observe and register (by photography) the fringes formation the models were submitted to a static load. The load was predetermined (0.5 Kgf) with a previous test with intention to concentrate the fringes formation until the order four. The models were placed in the circular polariscope associated with a cell load and axial and nonaxial load were applied with a universal testing machine (Lider, Araçatuba, São Paulo, Brazil) at fixed points in the central fossa of each prosthetic crown each time. The models were placed in a device with angle of 15° for nonaxial loading [3].

### 2.4. Analysis of Fringe Orders

To standardize the quantitative analysis of the fringe orders, 16 points were selected along the body of the two implants. These points were determined from photoelastic model images obtained by digital camera Cannon EOS T3i (Cannon USA, Inc., New York, NY, USA), using Fringes software in the MATLAB platform LPM/FEMEC/UFU. This experiment used quasi-tridimensional photoelastic analysis.

**Figure 3:**
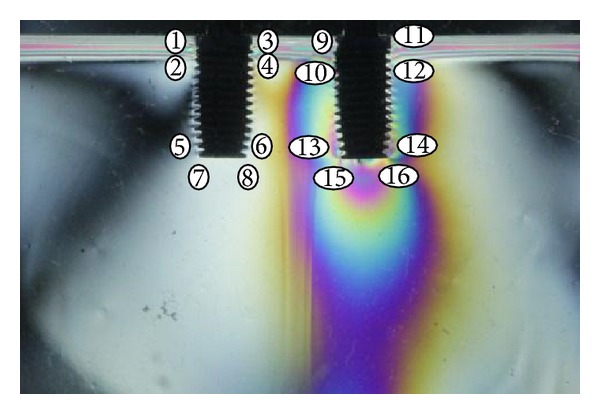
Representation of the points to be analyzed by Fringes software.

All models were analyzed using a template with dimensions of height and length of 25 × 50 mm. This technique made it possible to standardize the position of the points alongside the implants (Figure 3). For each point selected in every image, the isochromatic pattern orders of fringes (Nf) and the direction of the stress propagation were determined by consensus analysis performed by two examiners, using photoelastic resin colors scale. The stress shear was given by the Fringes program in the MATLAB platform LPM/FEMEC/UFU.

### 2.5. Statistical Analysis

Descriptive statistics were expressed as mean ± standard deviation (SD). Data were tested with Kolmogorov-Smirnov statistics for normal distribution. Levene's test was used to evaluate the homogeneity of variance. Data analysis was performed by three-way ANOVA to compare the factors implant length, crown height, and implant system. Post hoc comparisons were performed using Tukey's test (SAS v. 9.2; SAS Institute, Inc., Cary, NC).* P* values < 0.05 were considered significant.

## 3. Results

Overall data analysis showed that implant length was the only statistically significant factor for both axial (df = 1, *F* = 9.08, *P* = 0.003) and nonaxial loadings (df = 1, *F* = 10.94, *P* = 0.001). Short implants presented higher mean values of shear stress compared to long implants, regardless of load type. Interaction was observed between crown height and implant length (df = 1; *F* = 3.99; *P* = 0.047) and crown height and implant system (df = 1; *F* = 5.25; *P* = 0.023) for axial loading. However, for nonaxial loading, interaction was observed only between crown height and implant system (df = 1; *F* = 10.94; *P* = 0.001) (Tables 2 and 3).

The role of implant length in stress distribution was influenced by the crown height under axial loading. Short implants resulted in higher stress levels than long implants did when 8 mm prosthetic crowns were used (*P* < 0.05). However, this statistical difference in stress distribution was not observed with 13 mm prosthetic crowns (Table 4).

Short implants resulted in higher stress levels than long implants did when 8 mm prosthetic crowns were used (*P* < 0.05). However, this statistical difference in stress distribution was not observed with 13 mm prosthetic crowns (Table 4).

Another interaction under axial loading was observed between implant system and crown height. It is explained by the influence that the crown height exerted only on Neodent system. The 13 mm prosthetic crown showed significantly higher shear stress than 8 mm prosthetic crown in Neodent system implants (*P* < 0.05) (Table 4). In contrast, stress distribution in Bicon implants was not influenced by crown height.

Under nonaxial loading, Neodent implants with 8 mm prosthetic crowns resulted in mean stress values significantly higher than Bicon implants with the same crown height (*P* < 0.05). However, this difference in stress distribution between implant systems was not observed when 13 mm prosthetic crown was used (Table 5).

## 4. Discussion

Short implant indication is based on the principle that the transmission of biomechanical forces to the implants is concentrated in the cervical area [13, 14]. Thus, implant length could not be a significant factor in stress distribution. However, some trials show lower survival rates for shorter implants [15, 16]. Generally, this study showed that short implants resulted in higher stress shear than long implants did, regardless of the type of loading. Conversely, other factors—such as prosthetic crown height of fixed prostheses or implant macrostructure—did not influence the stress distribution when the whole data were considered. Therefore, implant length was the most important study factor. This finding may be related to the reduced surface area of the short implants, which can lead to greater stress concentration even with fixed prostheses, as used in this study. Current clinical studies have shown success rate for short implants similar to the success rate observed for long implants, ranging from 93.9% to 100% [17]. These findings can be explained by the increased area of bone-implant contact obtained by different surface treatments, different threads designs and types of implant prosthetic connection [6, 17]. The mentioned factors influence the concentration of cells involved in osseointegration.

Other methods to reduce biomechanical stress at bone-implant contact, as the use of fixed prostheses and absence of cantilever, are suitable approaches for dental implant therapy. In the case of short implants, Misch et al. [4] reported that fixed prostheses can enhance the predictability and success of rehabilitation of partially edentulous patients by increasing the number of implants and increasing the surface area on which the occlusal force is transmitted. However, our findings have shown that, even in this case of fixed partial prostheses, reduced implant length was a factor that induced increased shear stress around the implant. Thus, one can assume that, even in the face of prosthetic approaches to decrease the occlusal force transmitted to implants, the short length of implants can lead to higher stress concentration at the contact bone-implant and may contribute to biomechanical complications in dental implant therapy, as mentioned by Chang et al. [5]. This becomes clear when taking into account that the highest failure rate of short implants compared to longer implants was actually related to failures after prosthetic loading and not with the surgical technique and with the early osseointegration [4].

In a prospective clinical study with 36-month follow-up, Malchiodi et al. [6] showed that crown-implant ratio larger than 2 resulted in 96.6% success rate while the crown-implant ratio between 2 and 1.5 and less than 1.5 resulted in a success rate of 98.6% and 100%, respectively. The increase in crown height of 10–20 mm enhances by around 100% the amount of total force applied in a given implant system [18], which will be subjected to large flexion bending forces [3]. With such evidence, Neodent implants with 13 mm prosthetic crowns presented greater stress than Neodent implants with 8 mm prosthetic crowns, under axial loading. This reinforces the theory of lever arm related to the crown-implant ratio. Thus, Neodent implant system may not be indicated when the prosthetic rehabilitation of the patient results in increased crown height. Nonetheless, Bicon implants (short and long), under axial loading, behaved similarly, regardless of crown height. This fact can be explained by the tapered threads and positive neck collar macrostructure of these implants [19, 20]. Therefore, assuming increased crown height is a significant risk factor for implant failures [4], it seems rational to choose Bicon system implants in situations where prosthetic rehabilitation with increased crown height is necessary. According to Cehreli et al. [8] the macro- and microstructure of the implants may explain the mechanical behavior of dental implants. Patra et al. [19] reported that implants with a tapered thread design, such as Neodent system's threads, exhibited higher stress levels than implants with a parallel thread design do. Thus, Bicon system's parallel threads could have minimized the role of 13 mm prosthetic crowns as force magnifiers. However, our results are in contrast to those obtained by Chun et al. [21], with maximum stress levels found in the plateau-type thread configuration of Bicon system used in this study.

As previously mentioned, prostheses, with higher crown height, result in the magnification of the force transmitted to the implants [4]. In overload situations, there is an increase of deformation around the implants [11]. In this study, under axial loading, short implants had greater stress than long implants when 8 mm prosthetic crowns were used. This finding, however, was not observed when using 13 mm prosthetic crowns. So, in situations, in which the prosthetic crown results in overloading, it can be assumed that force magnification occurred in both short and long implants since the two implant lengths tended to show stress distribution in a similar way.

Under nonaxial loading, the difference in stress distribution between implant systems was influenced by the crown height. When 13 mm prosthetic crowns were used, both systems had the same behavior against the negative effect of increased prosthesis height and the lateral force. However, when 8 mm prosthetic crowns were used, Neodent implants resulted in higher levels of stress than Bicon implants did. Better results for Bicon implants can be explained both by the aforementioned thread design and by the configuration of the neck collar of Bicon implants. These findings suggest that the variations in the macrostructure of the implant to minimize the stress around the dental implant can have a significant impact when optimal prosthetic conditions are achieved. However, in prosthetic situations that increase the magnitude of force transmitted to the implants, such as increased prosthetic crown height, the effect of the geometry of the implant may not be significant.

It is known that lateral forces represent an increase of 50–200% in the force applied over implants as compared to vertical forces [4]. Thus, the nonaxial load applied in this study should also be considered as a factor that magnifies the forces transmitted to the implants. Nonetheless, nonaxial loading did not result in similar distribution of forces in the two implant systems studied. The explanation of this finding must take into account that nonaxial loading leads to higher stress level in the cervical area of the implant [22, 23]. Thus, the positive slope and cervical collar height of Bicon implants may have minimized the magnitude of the stress around the implant in this area, as mentioned by Faegh and Muftu [20].

## 5. Conclusion

Within the limits of the present study, it can be concluded that short implants concentrate larger stress levels than long implants do; however, when prosthetic factors that maximize stress around implants are present, as prosthetic crown with increased height, the implant system and implant length are not relevant factors.

## Figures and Tables

**Table 1:** Study design.

Group	Replicas	Implant characteristic	Load	Crown height (mm)
I	4	Neodent implant (long) 5 × 11 mm Titamax CM Cortical	Axial	8
				13
			Nonaxial	8
				13

II	4	Bicon implant (long) 5 × 11 mm	Axial	8
				13
			Nonaxial	8
				13

III	4	Neodent implant (short) 5 × 6 mm Titamax WS Cortical	Axial	8
				13
			Nonaxial	8
				13

IV	4	Bicon implant (short) 5 × 6 mm	Axial	8
				13
			Nonaxial	8
				13

**Table 2:** Three-way ANOVA for stress distribution under axial loading, based on the factors crown height, implant length, and implant system.

Source of variation	df	Sum of squares	Mean square	*F*	*P*
Crown	1	0.665	0.665	0.40	0.538
Implant length	1	15.103	15.103	9.08	0.003∗
System	1	0.195	0.195	0.12	0.733
Crown/implant length	1	6.633	6.633	3.99	0.047∗
Crown/system	1	8.726	8.726	5.25	0.023∗
Implant length/system	1	6.358	6.358	3.82	0.052
Crown/implant length/system	1	5.322	5.322	3.20	0.075

*Statistically significant difference.

**Table 3:** Three-way ANOVA for stress distribution under nonaxial loading, based on the factors crown height, length, and implant system.

Source of variation	df	Sum of squares	Mean square	*F*	*P*
Crown	1	0.504	0.504	0.29	0.593
Implant length	1	19.309	19.309	10.94	0.001∗
System	1	1.151	1.151	0.65	0.420
Crown/implant length	1	3.715	3.715	2.11	0.148
Crown/system	1	7.859	7.859	4.45	0.036∗
Implant length/system	1	0.888	0.888	0.50	0.479
Crown/implant length/system	1	6.397	6.397	3.63	0.060

*Statistically significant difference.

**Table 4:** Comparison of the study factors implant length and implant system relative to the crown height under axial loading in MPa.

Factor	Variables	Prosthetic crown	
		8 mm	13 mm
Implant length	Short	33.4 ± 16.0 Aa	28.3 ± 12.2 Aa
	Long	24.6 ± 12.5 Ba	26.8 ± 13.4 Aa

System	Neodent	25.7 ± 11.4 Aa	31.5 ± 16.5 Ab
	Bicon	26.5 ± 13.0 Aa	29.4 ± 13.9 Aa

*Note.* Different uppercase letters represent statistically significant differences between implant length and between implant systems. Different lowercase letters represent differences between crown heights (Tukey's test; *P* < 0.05).

**Table 5:** Comparison of the study factor implant system relative to the crown height under nonaxial loading in MPa.

System	Prosthetic crown	
	8 mm	13 mm
Neodent	31.43 ±15.44 Aa	28.13 ± 12.66 Aa
Bicon	26.18 ± 13.00 Ba	30.92 ± 14.89 Aa

*Note.* Different uppercase letters represent statistically significant differences between implant systems. Different lowercase letters represent differences between crown heights (Tukey's test; *P* < 0.05).
